# Numerical Simulation and Experimental Verification of Melt-Spinning Parameters’ Effects on Multi-Leaf Hollow-Profiled Fiber Preparation

**DOI:** 10.3390/polym16020228

**Published:** 2024-01-13

**Authors:** Shiqun He, Xinkang Xu, Pei Feng, Chongchang Yang, Shengze Wang

**Affiliations:** 1 College of Mechanical Engineering, Donghua University, Shanghai 201620, China; 1189709@mail.dhu.edu.cn (S.H.); 1212014@mail.dhu.edu.cn (X.X.); ycc@dhu.edu.cn (C.Y.); wasz@dhu.edu.cn (S.W.); 2 Engineering Research Center of Advanced Textile Machinery, Donghua University, Shanghai 201620, China

**Keywords:** multi-leaf profiled fiber, hollow fiber, profiled fiber, cross-section patterns, numerical simulation, melt-spinning process

## Abstract

Multi-leaf hollow-profiled fiber is a complex-shaped fiber with a hollow structure with at least three leaves arranged outside. In this work, spinning processes for the preparation of multi-leaf hollow-profiled fiber with complex cross-section patterns were proposed. Initially, the characteristics and preparation methods of multi-leaf hollow-profiled fibers were analyzed, and the key technologies for their preparation were studied. Further, micro-hole spinnerets were designed, and the numerical simulations of melt flow in the spinning channel were performed. Then, the preparation of six-leaf hollow profiled fibers was carried out to study the formation of the cross-sections. Finally, as an extension and application, an experimental verification of the melt spinning parameters’ effects on eight-leaf hollow fiber preparation was conducted. From the results of the spinning experiments, it was found that when the volume flow rate of a single hole increased from 2.33 × 10^−8^ m^3^/s to 3.33 × 10^−8^ m^3^/s, the profile degree of the spun fiber increased from 30.93% to a maximum value of 40.99%. Furthermore, when the cooling speed increased from 0.6 m/s to 1 m/s, the profile degree increased from 29.56% to 41.63%. When the initial blowing height increased from 80 mm to 140 mm, the profile degree decreased from 40.99% to 27.13%. When the spinning temperature increased from 285 °C to 290 °C, the profile degree decreased from 40.99% to 38.56%. However, the winding speed had an insignificant effect on the cross-sectional shape of the spun fibers. Moreover, the spun fibers showed good performance and a natural three-dimensional crimp function.

## 1. Introduction

The modification methods of fibers consist of physical methods and chemical methods. The wearability can be improved to a certain extent or even endowed with new features after modification, causing the products to vary significantly from standard fibers [1]. The preparation of profiled fiber is currently the most common technology for producing differential fibers. Presently, the main method of manufacturing profiled fiber is by changing the shape of the spinneret microhole [2]. Due to their high surface area, the moisture regain of the profiled fibers increases. The more complex the cross-section, the higher the moisture regain, thereby improving the wearability and increasing the composite properties [3,4]. The cross-section of the multi-leaf fibers usually presents three or more leaf-like branches. The common types of profiled fiber include three-leaf fibers, six-leaf fibers, cross fibers (i.e., four-leaf fibers), eight-leaf fibers, etc. The main features of such fibers is that their specific surface area is larger than that of the ordinary circular cross-section ones. Furthermore, the fiber surface is not only convex and grooved, but also distributed with numerous micropores. Moreover, the multi-leaf fibers are capable of achieving rapid water absorption, diffusion, and volatilization according to the capillary principle, effectively carrying moisture and sweat away from the skin surface and transmitting it to the outer layer of the fabric for evaporation. For example, the four-leaf fiber with four grooves named Topcool cannot hold water droplets stably. Other fibers, such as the Coolplus fibers with three-leaf and four-leaf cross-sections produced by Taiwan’s Zhongxing Textile Company, as well as the five-leaf polyester fibers with five longitudinal grooves developed by Quanzhou Haitian Light Textile Company, have the advantages endowed bycapillary phenomena, generated due to the microgrooves on the fiber surface for perspiration [5,6]. Gan Xuehui et al. conducted numerical simulations on the preparation process of the cross fibers and provided a theoretical basis for the development of cross-profiled fibers [7]. The functional cross fiber named Coolmax, with four grooves, has the advantages of excellent moisture absorption and quick drying performance [8]. Jung et al. conducted relevant research on the influence of the spinning conditions on cross-sectional changes in the three-leaf fibers. It was found that the shape factor of three-leaf fibers increased as the single-hole volume flow rate and winding speed increased when PET was used as the raw material [9].

Moreover, the stiffness of the fibers is also improved due to their hollow structure, endowing them with better bending and wear resistance [10,11]. The hollow shape has also evolved from the initial circular shape to other shapes such as triangles and quadrilaterals [12]. Twinair fiber, developed by AsahiKASEI, is a kind of special polyester hollow fiber with a hollowness of 30%, which has high water-absorbing quality compared with the other hollow fibers [13]. A new C-section hollow polyester filament produced in Japan can obtain a degree of hollowness up to 50% [14].

With the continuous improvement of people’s standards regarding their quality of life, new types of fibers with better functions have become a necessity for research and development. From the literature, it can be observed that high-simulation, multifunctional, and high-tech are the main trends [15]. It was also found that multi-leaf hollow fiber is a kind of fiber product that integrates multiple excellent properties. Esra Karaca et al. studied the cross-sections of four different fibers for their structure and properties, including three-leaf hollow fibers. They also conducted related studies on the thermal conductivity, heat absorption, and other properties of eight woven fabrics made of these four fibers [16,17]. Shen Xin et al. improved three-dimensional crimped hollow fibers using optimizing process conditions such as spinning, cooling, drawing, and crimping, successfully producing 6.0 dtex high-quality hollow polyester fibers similar to natural furs, with properties such as good thermal insulation and high resilience [18]. Cao Qiang et al. found that triangular hollow structures have stronger deformation resistance and a more stable structure when compared to circular hollow structures. They also have a relatively larger specific surface area, a softer texture, better anti-pilling properties, stronger compression resistance, and superior performance in terms of deformation resistance [19]. By using three-hole hollow fibers, Chen et al. developed durable Janus hollow fibers that have been gradually developed and applied in many fields in recent years due to their unique characteristics [20]. Taehwan Oh et al. used the finite element method to simulate the melt-spinning process of hollow fibers. Considering the influence of spinning process conditions and microhole geometry on the spinning process, they established dynamic formulae for the spinning of hollow fibers [21,22,23]. Rebecca et al. found that both the processing conditions and the die wall thickness affect the hollowness of the hollow fibers [24,25]. Yang et al. conducted preparation experiments on 15-hole and 17-hole square hollow polyester fibers [26]. Presently, research on multi-leaf hollow profiled fibers is widely produced and applied both nationally and internationally, and it can be observed that there is a lack of relevant theoretical research and practical experience. 

In this paper, preparation methods for multi-leaf hollow profiled fibers based on complex cross-section shapes and structure were studied. According to the design requirements of the melt spinning process, the simulation of multi-leaf hollow structures was carried out. Furthermore, melt spinning experiments were conducted on six-leaf hollow profiled fibers used as the raw material of PET, and then changes in cross-sections based on the experimental results were analyzed. In order to verify the preparation methods proposed, eight-leaf square hollow profiled fibers with excellent performance were developed, and fiber preparation experiments were also conducted to investigate the influence of spinning process parameters on the cross-sectional shape of the fibers.

## 2. Formulations and Numerical Simulations

**Figure 1:**
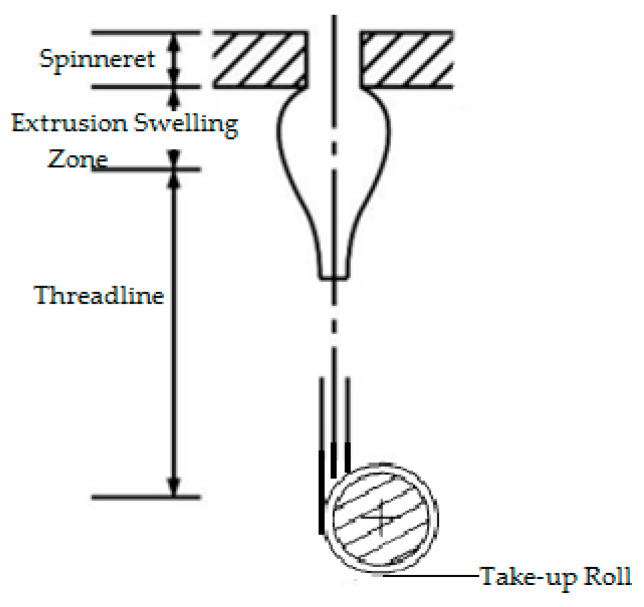
Schematic representation of the melt-spinning process.

In this work, the process of melt spinning is applied to extrude a polymer melt continually through the spinning pack. After extrusion from spinneret micro-holes, the melt flow will be stretched along the threadline. Simultaneously, extrusion swelling in the polymer melt occurs, and energy exchange occurs via the process of extrusion [27]. Further, fibers are mainly classified based on changes in the fluidity and cross-sections of polymer melts during the melt-spinning process, as Figure 1 shows.

The polymer melt is susceptible to radial and transversal velocity when fluids enter into spinneret holes, and these two different velocity gradients will cause elastic deformation due to the influence of the rheological state on the micro-hole passage. As a result of the die-swell effect, there is a significant difference between the cross-sectional shape and the spinneret hole after the polymer melt leaves the spinneret. After this process, the polymer melt will be elongated and cooled in the direction of the threadline, and will ultimately undergo winding formation. These are the major phases of the cross-section changes. Therefore, multiple factors can affect the shapes of the cross-sections of profiled fibers, which includes melt viscosity, melt surface tension, cooling conditions, drawing ratio, etc. 

### 2.1. Dynamic Model for Spinning of Profiled Fibers

Considering the characteristics of polymer melt and the conditions for stable extrusion, the following assumptions are made: (a) it involves steady-state spinning, (b) it has constant physical properties, (c) viscous heat dissipation is neglected, (d) it is subjected to negligible effects of inertial force and gravity, and (e) the normal and tangential velocities are equal to zero in the hole wall. For any shape of the cross-section, during non-compressible viscoelastic fluid extrusion, the continuity equations of mass conservation, momentum conservation and energy conservation [28] for the melt spinning system can be written as shown in Equation (1).

(1) $$\frac{\partial \rho }{\partial t}+\frac{\partial (\rho u)}{\partial x}+\frac{\partial (\rho v)}{\partial y}+\frac{\partial (\rho w)}{\partial z}=0$$

In Equation (1), ρ refers to the density of the melt, *t* refers to the time, and *u*, *v*, and *w* represent the partial velocities on the *x*, *y*, and *z* axes, respectively. All fluids flowing continuously should also comply with the momentum conservation equation, which is shown in Equation (2).

(2) $$\frac{\partial \left(\rho u_{x}\right)}{\partial_{t}}+\nabla •\left(\rho u_{x}\vec{u}\right)=-\frac{\partial p}{\partial x}+\frac{\partial \tau_{xx}}{\partial x}+\frac{\partial \tau_{yx}}{\partial y}+\frac{\partial \tau_{zx}}{\partial z}+\rho f_{x}\;\frac{\partial \left(\rho u_{y}\right)}{\partial_{t}}+\nabla •\left(\rho u_{y}\vec{u}\right)=-\frac{\partial p}{\partial y}+\frac{\partial \tau_{xy}}{\partial x}+\frac{\partial \tau_{yy}}{\partial y}+\frac{\partial \tau_{zy}}{\partial z}+\rho f_{y}\;\frac{\partial \left(\rho u_{z}\right)}{\partial_{t}}+\nabla •\left(\rho u_{z}\vec{u}\right)=-\frac{\partial p}{\partial z}+\frac{\partial \tau_{xz}}{\partial x}+\frac{\partial \tau_{yz}}{\partial y}+\frac{\partial \tau_{zz}}{\partial z}+\rho f_{z}$$

In Equation (2), p refers to the pressure on the surface of the fluid unit, $\tau_{xx}$ , $\tau_{yx}$ , $\tau_{zx}$ , $\tau_{xy}$ , $\tau_{yy}$ , $\tau_{zy}$ , $\tau_{xz}$ , $\tau_{yz}$ and $\tau_{zz}$ are the viscous stress components of the molecular viscosity effect acting on the surface of the unit, and $f_{x}$ , $f_{y}$ and $f_{z}$ are the unit mass forces on three coordinate axes. The energy conservation equation shows that the total amount of energy is constant in the system, which can be defined as shown in Equation (3):

(3) $$\frac{\partial \left(\rho E\right)}{\partial_{t}}+\nabla •\left[\vec{u}\left(\rho E+p\right)\right]=\nabla •\left[k_{eff}\nabla T-\sum_{j}h_{j}J_{j}+\left(\tau_{eff}•\vec{u}\right)\right]+S_{h}$$

In Equation (3), E represents the total energy of the unit, *h* represents enthalpy, $h_{j}$ represents the enthalpy of component *j*, $k_{eff}$ is the effective thermal conductivity coefficient, $\tau_{eff}$ is the turbulent thermal conductivity coefficient, $J_{j}$ represents the diffusion flux of each component *j*, and $S_{h}$ refers to customized volumetric heat source terms, such as chemical reaction heat.

The constitutive equation, known as the rheological equation, is usually employed for the description of the relationship between stress and strain rates. The constitutive model of materials is applied in relation to the equation indicating the relationship between the deviatoric stress tensor and the strain rate tensor of viscoelastic fluid flow [29]. The Bird Carreau model can be employed to demonstrate the viscosity characteristics of movements with low shear rates, as shown in Equation (4).

(4) $$\eta =\eta_{\infty }+(\eta_{0}-\eta_{\infty }){\left(1+\lambda^{2}\dot{\gamma^{2}}\right)}^{\frac{n-1}{2}}$$

In Equation (4), η refers to the melt shear viscosity, $\eta_{\infty }$ refers to the viscosity at an infinite shear rate, $\eta_{0}$ refers to zero shear viscosity, λ refers to the relaxation time, and n refers to the non-Newtonian exponent.

### 2.2. Deformation Dynamics of Cross-Section of Profiled Hollow Fibers

The shapes of profiled spinneret holes can be considered as composites with many rectangular slits that can be arranged as required, and the relevant equation can be deduced theoretically [30]. The expression is shown in Equation (5).

(5) Δτ=ΔE

The left side of the equation represents the energy difference that arises due to the decreased surface area of the melt within a unit of time, while the right side of the equation represents the energy dissipation that occurs during the flow process in a unit of time. After calculation and derivation, Equation (6) can be obtained [30].

(6) $$\frac{dk}{\left(k^{2}-1\right)}=-\frac{F_{s}}{2\eta \sqrt{A}\;V}dz$$

In Equation (6), *k* is the deformation coefficient, *F_s_* refers to surface tension, *A* refers to the cross-sectional area of the fiber, *V* refers to the flow velocity of micro-segments, and *η* refers to the extensional viscosity of the melt.

Assuming a constant cross-section area ( ${A_{i}=A}_{i+1}$ ) of about Δz micro-section, we selected a value of the radius of r(i) between the outer radius $R_{0}(i)$ and inner radius $R_{i}(i)$ to satisfy the following equation [31]:

(7) $$\frac{d\pi \left({R_{0}}^{2}-{R_{i}}^{2}\right)}{dt}=0$$

**Figure 2:**
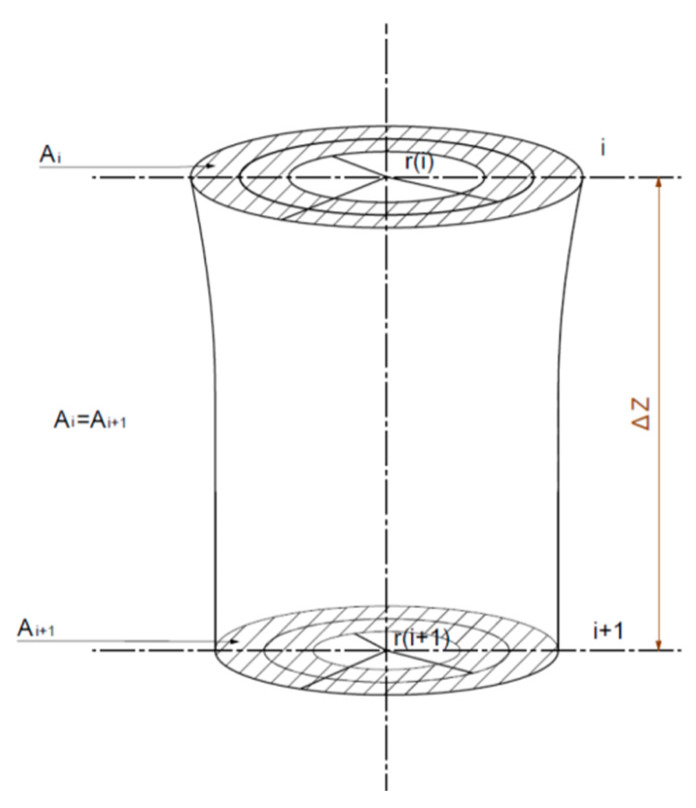
Cross-section changes in micro-section of hollow fibers arising in the spinning process.

After passing through the micro-section Δz in the direction of the spinning process, the value of the radius r(i) becomes r(i+1) , as shown in Figure 2 [31]. Via derivation and error analysis, according to Equation (7), the hollowness B(i) of the hollow fiber in any segment can be determined as shown in Equation (8).

(8) $$B(i)=\frac{\pi {\left(R_{i}+\Delta R_{i}\right)}^{2}}{A_{i}+\pi {\left(R_{i}+\Delta R_{i}\right)}^{2}}$$

To ensure the even and continuous extrusion of the tow, it is necessary to ensure that the polymer melt is uniformly distributed at each micro-hole, enabling one to achieve a consistent pressure drop across all channels. Further, the design of the filter layer should be constructed so as to guarantee the filtration’s accuracy and ensure the good homogeneous effect of the melt. Moreover, the pressure applied through the filter layer should increase slowly in order to guarantee a relatively long continuous working time [32,33,34]. The empirical calculation formula for the pressure drop encountered in the filter screen is shown in Equation (9).

(9) $$\begin{array}{c} \Delta P=\frac{64{\left(D_{0}+D_{S}\right)}^{2}D_{S}\mu Q}{FD_{0}^{4}}\\ F=ZD_{0}^{2} \end{array}$$

In Equation (9), ΔP refers to the pressure drop, *Q* refers to the total volume flowing through the filter, *D*_0_ refers to the spacing between the holes in the filter mesh, *D_s_* refers to the diameter of the filter mesh, *μ* refers to the viscosity of the melt, *F* refers to the total through-hole area of the filter, and *Z* refers to the number of filter holes.

The formula for the pressure drop in the distribution board is shown in Equation (10).

(10) $$\Delta P=\frac{128QL\eta }{\pi Z_{0}d^{4}}$$

The formula for calculating the average flow velocity of a single hole is shown in Equation (11).

(11) $$v=\frac{4Q}{\pi Z_{0}d^{2}}$$

In Equation (11), η refers to the fluid viscosity, *Q* refers to the total inlet flow rate of the melt, *L* represents the depth of the flow channel, *d* refers to the hole diameter, and $Z_{0}$ is the number of holes in the distribution board.

### 2.3. Design of Spinneret

The design steps for the spinneret are as follows: Initially, the number of leaves of the multi-leaf hollow profiled fibers have to be determined, i.e., the number of rectangular slits and the number of segments of the circular slits that make up the hollow structure. Then, rectangular and circular slits are arranged in a uniform pattern. During the design, it is necessary to consider the proportional relationship between the number of rectangular slits and the number of circular slits in order to make sure each complex unit is relatively consistent in shape. Furthermore, in order to maintain the good cross-sectional shape of the protruding leaves, rectangular slits are modified into trapezoidal slits.

**Figure 3:**
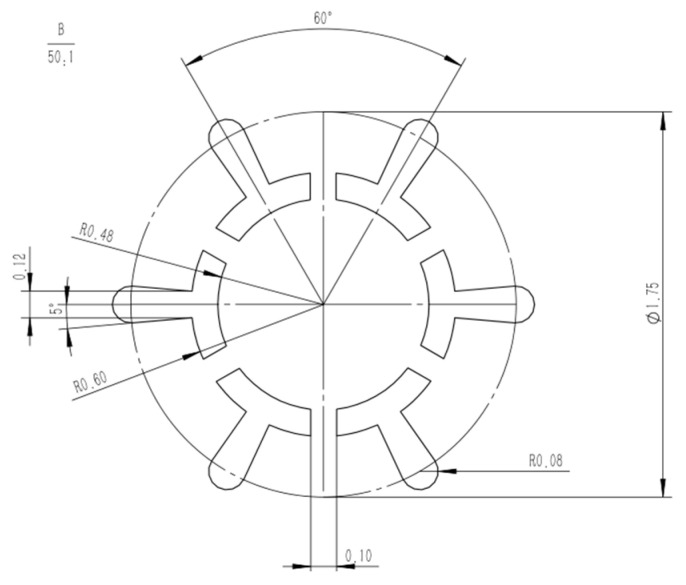
Micro-hole design diagram of six-leaf hollow profiled fibers.

Six-leaf hollow shaped fibres and three-leaf hollow profiled fibres are considered two typical types of shaped fibres, with six-leaf hollow fibres having more blades than three-leaf hollow shaped fibres. Furthermore, the symmetrical distribution of the six-leaf hollow fibre makes them appear more complex. Therefore, in this work, a spinneret with six-leaf hollow micro-hole shapes was designed to study the cross-sectional formation of multi-leaf hollow profiled fibres. The micro-hole shape is shown in Figure 3.

Further, the micro-hole design size of the spinneret for the six-leaf hollow profiled fibers is calculated based on the formula given in Equations (8)–(11). As the micro-hole shape is composed of multiple complex units, an independent complex unit for each hole is used to calculate the equivalent diameter and shear rate. Some of the parameters used during the simulation are listed in Table 1.

From the results shown in Table 1, it is clear that the equivalent diameters of the complex units of micro-holes are within the generally encountered range of 0.15–0.4 mm during melt spinning, and the corresponding shear rate is also lower than the critical shear rate of 10^4^ m/s. This has been found to meet the requirements of normal spinning.

### 2.4. Numerical Simulation of Melt Flow

In this section, appropriate material flow characteristic parameters and boundary conditions based on experimental experience are selected for the numerical simulation of the melt flow in the channel. In this study, a geometric model of the spinneret channel of the multi-leaf hollow shaped fiber is designed, and then converted into a suitable format and imported into simulation software. Further, the inlet volume flow rates for holes are set as shown in Table 2. To simplify the calculation and obtain reasonable results for simulation, the following assumptions are made based on technological processes and material characteristics: (1) the melt is isothermal; (2) the melt flow is an incompressible non-Newtonian fluid; (3) the melt flow type is non-slip flow in the spinneret channel; (4) the influence of inertial force and gravity is neglected. The Bird–Carreau constitutive equation is selected as the constitutive equation for polymer materials. The rheological parameters of PET materials used in the numerical simulation are shown in Table 2.

The melt inlet condition is the inlet volume flow rate for different holes based on the single-hole volume flow rate, as shown in Table 1.

The slip between the melt and the hole wall is ignored, and the normal and tangential velocities are taken as zero.

The melt outlet condition is that the normal stress and tangential stress are equal to zero.

Furthermore, in order to determine the pressure distribution, shear rate distribution, velocity distribution, and extrusion swell, numerical simulations have been conducted. The results are as follows:

(1) Simulation results of pressure distribution

**Figure 4:**
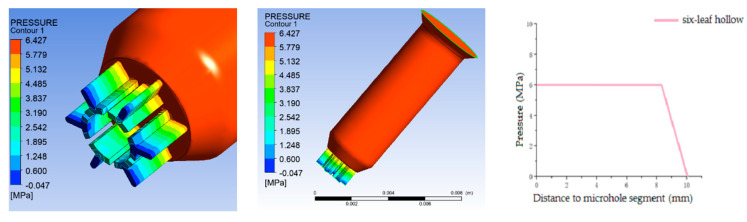
Pressure distribution of six-leaf hollow micro-holes.

Figure 4 shows the pressure distribution of six-leaf hollow micro-holes. It can be observed that when the melt is in the spinneret channel, the pressure undergoes a decreasing trend along the axis of the channel. It is also found that as the melt approaches the micro-hole segment, the pressure amplitude increases. The spinneret holes have significant impacts on the pressure loss in the channel, as shown in Figure 4.

(2) Simulation results of shear rate distribution

**Figure 5:**
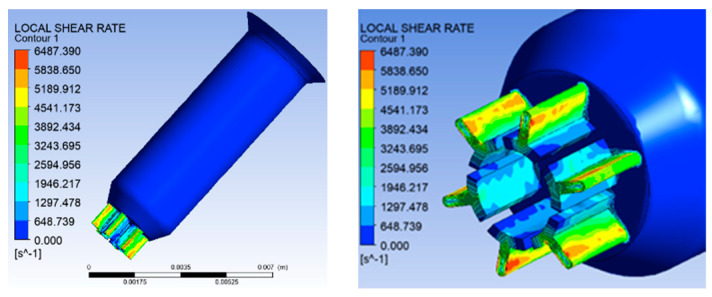
Shear rate distribution of six-leaf hollow micro-holes.

Figure 5 shows the shear rate distribution of six-leaf hollow micro-holes. From Figure 5, it can be observed that the maximum shear rate during melt flow occurs on the microporous wall. In addition, the maximum shear rate of 6487.39 s^−1^ occurs near the junction of the trapezoidal slits and the end arcs.

(3) Simulation results of velocity distribution

**Figure 6:**
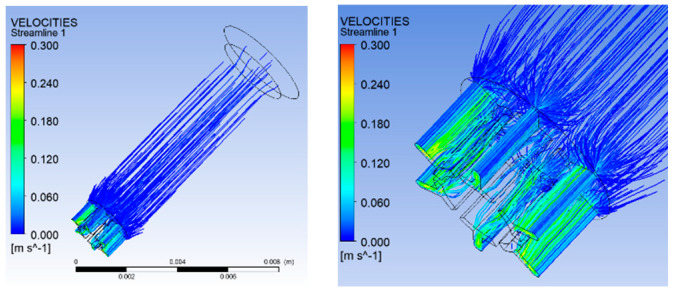
Distribution of overall velocity and micro-hole segment velocity.

Figure 6 shows the distribution of overall velocity and micro-hole segment velocity. From Figure 6, it can be observed that the flow velocity of the melt near the wall is relatively small. The maximum velocity of melt flow is 0.3 m/s, which appears in the microporous segment. Moreover, the maximum speed occurs in the trapezoidal slit of the leaf section.

(4) Numerical simulation of the extrusion swell

**Figure 7:**
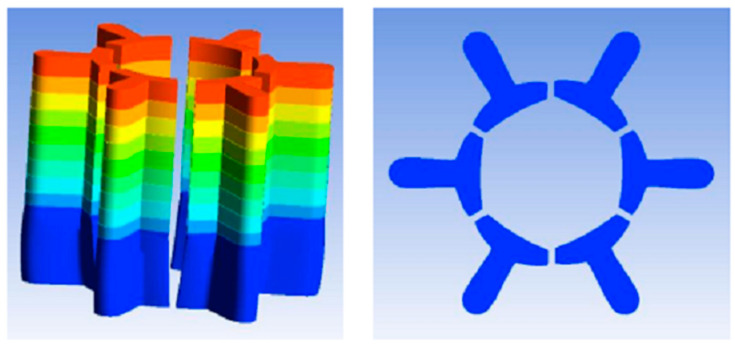
Extrusion swelling of six-leaf hollow profiled fibre micro-holes.

In the numerical simulation process, a geometric model of melt extrusion was established, and material flow characteristic parameters and corresponding boundary conditions were selected, as shown in Table 2. In addition, numerical simulations were conducted on the extrusion process. It was observed that the melt could not bond together within a range of 0.5 mm from the outlet of the spinneret after extrusion. However, it was found that the size of the gap is constantly narrowing. By judging the cross-sectional shape at a distance of 0.5 mm from the spinneret outlet, it was found that the contour of the hollow structure is composed of six larger arcs, similar to a regular hexagon. After forming a hollow structure, it was observed to deform into a circular hollow shape. It was also found that the change in leaf profile begins at the junction of trapezoidal and circular slits, which helps to form a better leaf profile. As shown in Figure 7.

## 3. Materials and Experiment

This section mainly discusses the equipment, materials, and related spinning process parameters used for the preparation of six-leaf hollow profiled fibers.

### 3.1. Fiber Preparation Equipment

**Figure 8:**
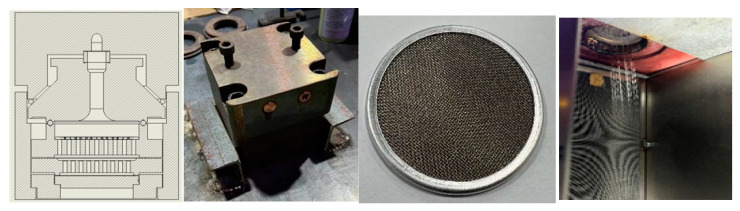
The spinning equipment for the six-leaf hollow fibers.

In this work, a six-leaf hollow cross-section spinning pack and spinning equipment were developed (Figure 8). Here, the melt flows into the pressure plate and then enters the sand cup. After being filtered and homogenized by the filter screen, the melt reaches the distribution plate, is evenly distributed on the spinneret plate, and is then extruded through the spinneret channel. Moreover, the spinning pack has an independent module that controls the melt flow in each component and makes technical adjustments to meet the spinning process’ requirements. For instance, the single-hole volume flow rate and spinning temperature can be adjusted by regulating the metering pump speed and the spin manifold temperature. 

Further, the pressure drop in the component is calculated based on the formula provided in the previous section (in Equations (9)–(11)). The pressure drop of the spinneret is obtained through numerical simulation, as shown in Table 3.

### 3.2. Spinning Process Parameters

The raw material used is ordinary PET chips (batch number: CT01). It is observed that moisture can reduce the viscosity and spinnability of polyester chips. Therefore, before spinning, the polyester chips are placed in a vacuum drum drying oven for 42 hours based on a pre-established program. After drying, they are transported to a screw extruder (manufacturer: Shuihong, Jingjiang City, Zhejiang Province, China) and heated for extrusion at a certain temperature.

**Figure 9:**
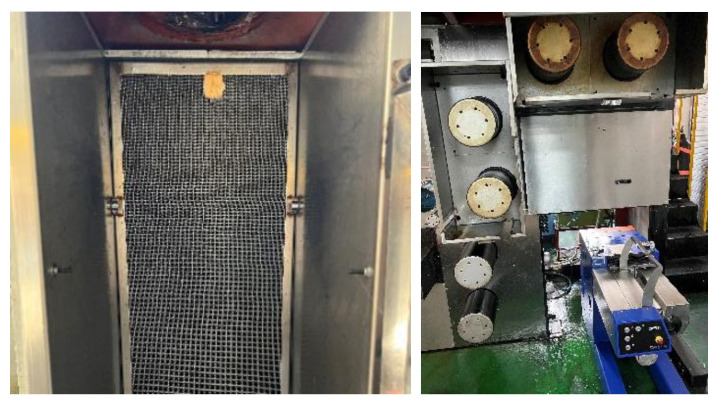
Cooling and winding device.

Figure 9 shows the cooling and winding device used in this work. During the extrusion forming process, the cooling speed and the initial blowing height are the key parameters. Besides this, when the winding speed is adjusted, the fineness of the fibers will also change, which has a certain impact on the formation of the cross-section. Therefore, it is necessary to control the temperature of the screw, the temperature of the melts, the temperature of the components, and the cooling speed during the spinning process.

### 3.3. Melt Spinning

An experiment on the preparation of six-leaf hollow profiled fibers was conducted according to the spinning process parameters proposed, as shown in Table 4.

**Figure 10:**
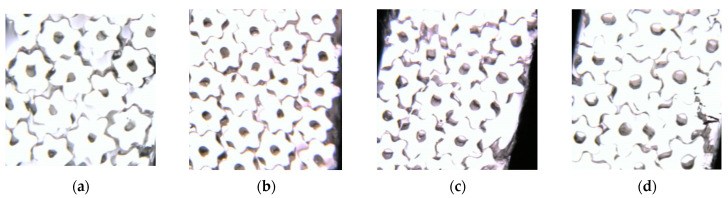
Cross-sections of six-leaf hollow profiled fibre with different cooling rates: (**a**) 0 m/s; (**b**) 0.3 m/s; (**c**) 0.6 m/s; (**d**) 0.9 m/s.

To observe the cross-sectional pattern of the fibres, a Hastelloy fibre slicer was used to slice the spun fibres. In addition, using an optical microscope to observe and record fibre cross-sectional patterns. Different cross-sectional shapes of fibres obtained at different cooling rates are shown in Figure 10.

From Figure 10, it can be observed that the cross-sectional shape of the spun fiber was formed effectively due to its relatively high extrusion speed during the preparation. Further, it is also observed that, as the cooling speed increased, the leaf profile and hollow structure formed more effectively, which indicates that cooling conditions have a positive correlation with the cross-section’s formation. It was found that the cross-sectional shape formed better at a cooling rate of 0.9 m/s.

## 4. Application and Results

Due to the requirements set regarding the symmetry of the cross-sectional shape of square hollow structures, an eight-leaf square hollow structure was chosen for this application. In this section, numerical simulation was applied to design the eight-leaf square hollow profiled spinneret hole, and the formation law of the cross-section was also studied based on the experimental results.

### 4.1. Design of Spinneret Micro-Hole for Eight-Leaf Square Hollow Profiled Fiber

**Figure 11:**
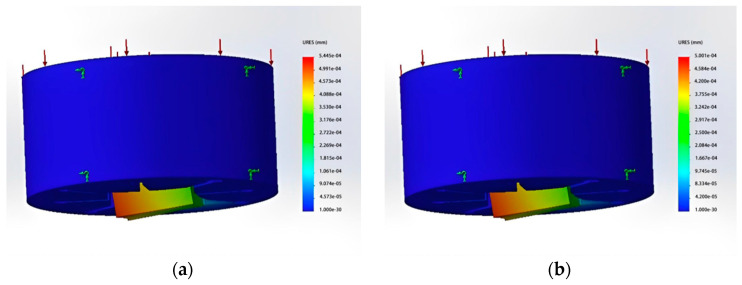

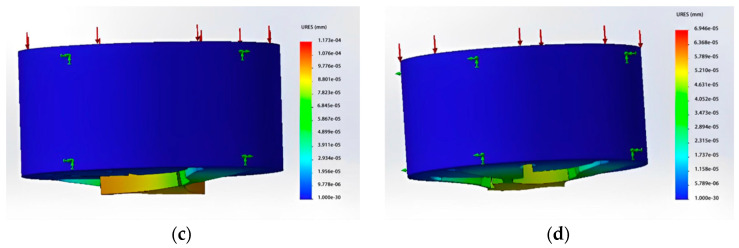
Total displacement results of static stress analysis. (**a**) The number of gaps is 1, and the gap dimension is 0.08 mm; (**b**) The number of gaps is 1, and the gap dimension is 0.1 mm; (**c**) The number of gaps is 2, and the gap dimension is 0.08 mm; (**d**) The number of gaps is 4, and the gap dimension is 0.08 mm.

In this work, the design method of spinneret orifice clearance space for six-leaf hollow is adopted to form a square hollow structure. Furthermore, convert the large circular arc slit into four uniformly distributed circular arc slits centered around the microholes.Moreover, the radius size of the arc segment slit is optimized based on the concave curve radius of the triangular profiled microholes. The number of complex units is divided into 1, 2, and 4 respectively so that a smooth square hollow structure can be formed. The number of partitions and gap dimensions are determined based on stress analysis. Stress analyses of micro-holes are divided into four groups, as shown in Figure 11. Dividing multiple units into 1, 2, and 4 can form a smooth square hollow structure. The number of partitions and gap dimensions are determined based on stress analysis. The stress analysis of micropores is divided into four groups, as shown in Figure 11.

**Figure 12:**
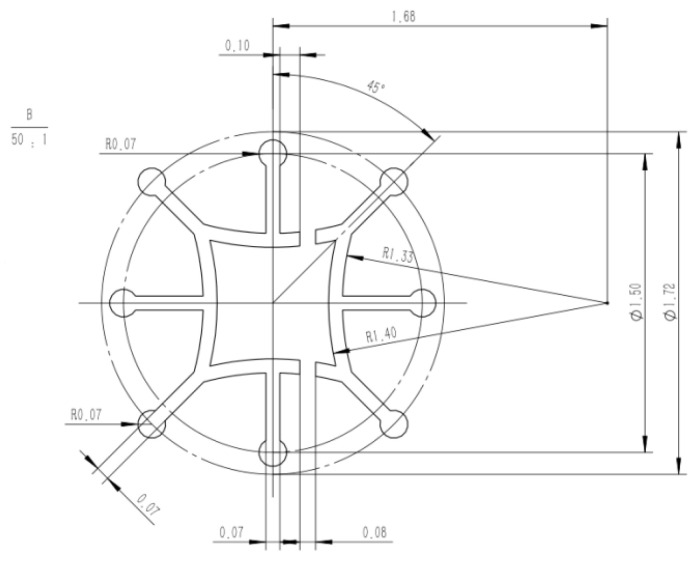
Micro-hole design diagram of an eight-leaf square hollow profiled fibre.

From the results of the stress analyses, it can be observed that when using a single-micro-hole design, the board surface forms a hollow structure and has only one support point. However, the position of the support point will be offset when subjected to high pressure. The maximum displacements of the board surface under these two types of stress are approximately 0.0005445 mm and 0.0005 mm, respectively. This indicates that using only one gap cannot significantly prevent deformation. When the gap dimension was set as 0.08 mm and the lower surface was divided into two and four micro-holes, it was found that the maximum displacement distances were about 0.00009 mm and 0.00005 mm. This is a better effect than was achieved by a single micro-hole in the surface plate. The eight-leaf square hollow spinneret used here has a fineness of 8D and is designed to be composed of two complex units. A schematic diagram of the micro-hole’s design is shown in Figure 12.

The modes of calculation of equivalent diameter, shear rate, and other parameters are shown in Table 5.

### 4.2. Numerical Simulation of Melt Flow

During the numerical simulation of the melt flow, the materials and other parameters used were the same as those applied in the previous section, and the results have been analyzed as follows:

(1) Pressure distribution

**Figure 13:**
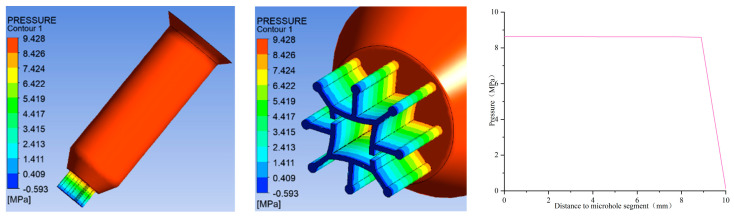
Pressure distribution of eight-leaf square hollow micro-holes.

From Figure 13, it can be observed that when the melt flows through the channel of the spinneret, the pressure undergoes a trend of decreasing only along the axis of the channel. The highest value of pressure drop was 9.428 MPa, and the change rate of pressure began to increase as it approached the micro-hole channel.

(2) Shear rate distribution

**Figure 14:**
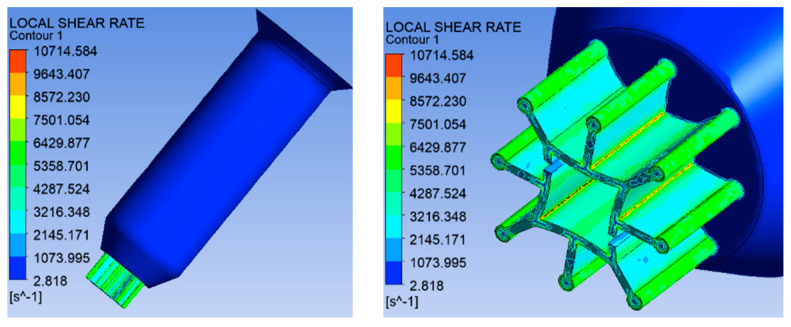
Shear rate distribution of eight-leaf square hollow micro-holes.

From Figure 14, it can be observed that the maximum shear rate of melt occurred on the wall of the micro-hole channel, with a maximum value of 10,714.584 s^−1^. It was also found that if the deformation first occurs in the area where the maximum shear rate is located, it can promote the formation of square hollow shapes to some degree.

(3) Numerical simulation of the extrusion swell

**Figure 15:**
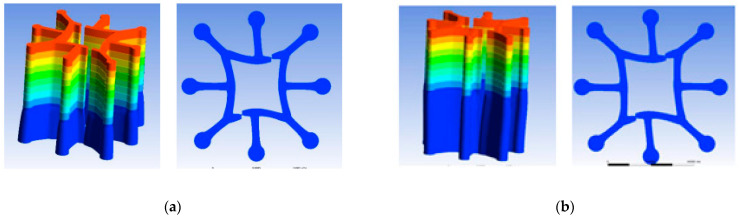
Extrusion swell of eight-leaf square hollow micro-holes. (**a**) 0.5 mm from the outlet of the spinneret (**b**) 1 mm from the outlet of the spinneret.

From Figure 15, it can be observed that when the melt was extruded at a distance of 0.5 mm from the outlet of the spinneret, the gaps narrowed significantly, but were not been bonded together. However, when the melt was extruded at a distance of 1 mm from the outlet of the spinneret, the four concave arcs bonded together. At the same time, the four segments gradually underwent deformation. As the radius increased, the arc came to more closely resemble a straight line, causing the shape of the hollow structure to tend towards a square.

### 4.3. Results and Analysis

**Figure 16:**
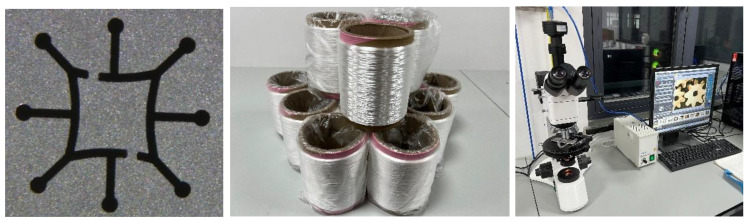
Preparation of eight-leaf square hollow profiled fibres.

Similarly to the eight-leaf square hollow profiled fiber spinneret designed with a filament size of 144D/18f, the preparation of the eight-leaf square hollow profiled fibers was carried out according to the spinning process parameters set for each group, as shown in Figure 16.

In order to observe the cross-section’s shape, a Y172 fiber slicer was used to slice the spun fibers that had been obtained, and the fiber’s cross-section morphology was observed and recorded using a microscope. The spinning temperature parameters were individually set using the spinning machine as listed in Table 6, and the influences of single-hole volume flow rate parameters were analyzed for their impact on the formation of the cross-section during preparation. 

From the results, it is observed that as the volume flow rate of a single hole increases, the overall degree of profile of the cross-sectional shape of the spun fibres increases initially and then decreases later. Accordingly, the contour of the leaf shape and the square hollow are found to be presented clearly initially and then blurred later. When the volume flow rate increases from 2.33 × 10^-8^ m^3^/s to 3.33 × 10^-8^ m^3^/s, the degree of profile increases from 30.93% to 40.99%, and then decreases to 36.7% when the volume flow rate increases to 4.33 × 10^-8^ m^3^/s.

Furthermore, the shape of the hollow fiber also changed from circular to square with the increase in the single-hole volume flow rate, with rates of 3.33 × 10^−8^ m^3^/s and then 3.67 × 10^−8^ m^3^/s. Finally, the contours of the square hollow shape were found to be more obvious, and they returned from square to circular as the volume flow rate of the single hole increased.

**Figure 17:**
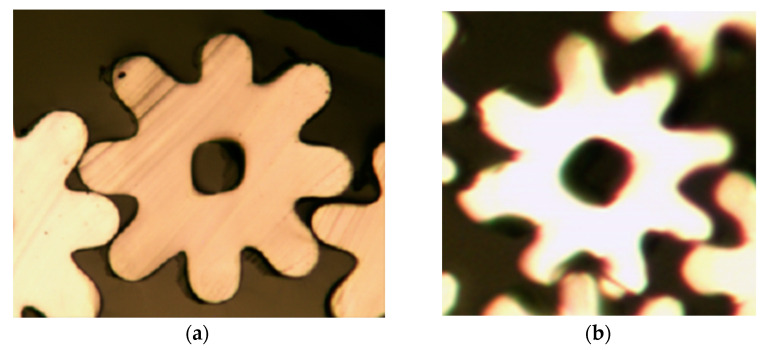

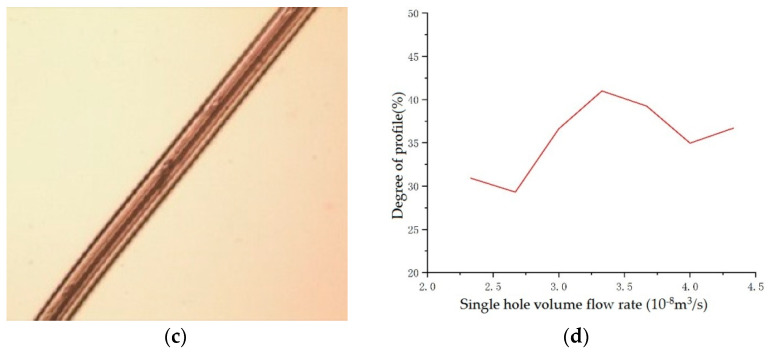
Single hole volume flow rate is 3.33 × 10^−8^ m^3^/s. (**a**) Spun fibre cross-section; (**b**) cross-section of fibre after winding; (**c**) fibre surface after winding; (**d**) Volume flow rate—degree of profile -transformation diagram.

From Figure 17, it can be observed that when the single-hole volume flow rate increases, the fiber’s fineness will be improved. Moreover, it will also increase the extrusion speed of the melt, allowing it to be exposed more quickly for cooling and thus slowing down the deformation of the cross-sectional shape. However, when the volume flow rate of a single hole is great enough, the fiber’s fineness also becomes larger, which leads to a longer cooling time and a corresponding increase in the time of deformation. As shown in Figure 17d, when the volume flow rate of a single hole was set to 3.33 at 10^-8^ m^3^/s, the degree of the profile reached optimality. Furthermore, the effects of the cooling rate, the initial blowing height, the spinning temperature, and the winding speed on cross-sectional deformation were also studied. The conclusions derived are as follows (the processes and results can be found in Supplementary Material part S1).

The cooling rate was increased from 0.6 m/s to 0.8 m/s and 1.0 m/s. As the cooling speed increased, the degree of the profile increased from 29.56% to 41.63%. Further, the hollow shape remained square with the increase in wind speed, causing the melt to cool faster. Moreover, the viscosity of the melt increased with the decrease in temperature, increasing the resistance of the fiber cross-section to further deformation. However, it was observed that when the cooling rate reached 1.0 m/s, some fibers were blown away from the nozzle during spinning; therefore, the optimal cooling air speed was inferred to be 0.8 m/s.

The parameters set for the initial blowing height were 80 mm, 110 mm, and 140 mm. It was found that as the blowing height increased, the cross-sectional shape of the spun fibers changed from a square hollow contour to a circular hollow contour, and the clarity of the outer leaf contour was also reduced. Further, the degree of the profile decreased from 40.99% to 27.13%. Moreover, as the initial blowing height increased, it took more time for the melt to come into contact with the cooling air after being extruded, and the time required for cross-sectional changes also increased. This had an adverse effect on the formation of the cross-section of the eight-leaf square hollow fiber.

Furthermore, experiments were conducted at spinning temperatures of 285 °C, 287 °C, and 290 °C, respectively. The results show that the cross-section of the spun fibers gradually deviated from the expected shape (within a certain range) as the spinning temperature increased, and the degree of the profile decreased from 40.99% to 35.86%. When the spinning temperature was 290 °C, the hollow outline of the spun fibers was not square in shape.

The change in winding speed had a negligible effect on the cross-section’s shape. However, when comparing between before winding and after winding, it was found that winding is conducive to the formation of the fiber’s cross-section. The final spinning process parameters obtained are shown in Table 7.

### 4.4. Performance Testing

The results of the mechanical performance testing and the testing of the natural 3D crimp characteristics are discussed below.

(1) Mechanical performance testing

**Figure 18:**
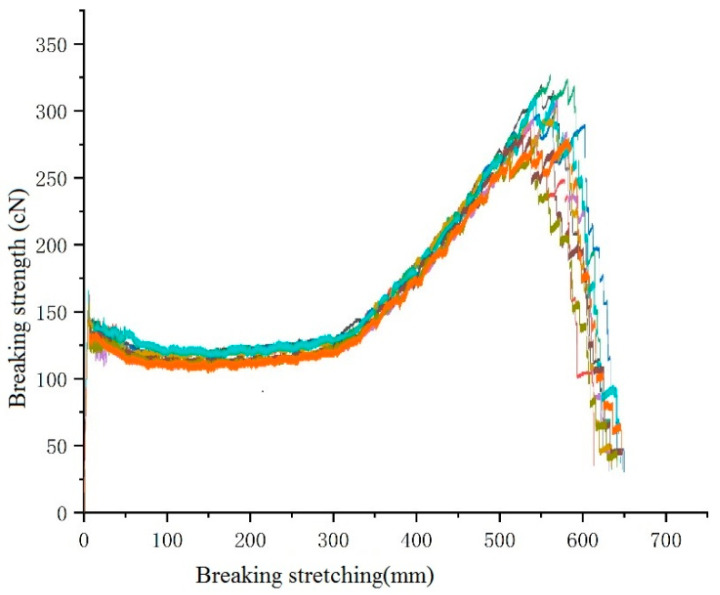
The tensile strength diagram of eight-leaf square hollow profiled fibres.

Initially, the mechanical properties of the fibres were tested. The spinning process parameters are as follows: The single hole volume flow rate was taken as 3.33 × 10^−8^ m^3^/s. The mechanical properties of eight-leaf square hollow fibres with a cooling speed of 0.8m/s were considered. An initial blowing height of 80 mm, a spinning temperature of 285 °C, and a winding speed of 1200 m/min were considered during the testing. The measurement results are shown in Figure 18. Different breaking strength are in different colour, related data and parameters can be seen in Supplementary Material part S2. 

From the results, it can be observed that the average tensile strength of an eight-leaf square hollow profiled fiber is 301 cN and its average elongation at break is 551.30%. In addition, if used in actual production, the technical parameters of stretching of this fiber must be optimized. Further, after stretching elongation exceeds 300%, the strength of the fibers increases rapidly with the increase in the stretching elongation. Furthermore, when the stretching elongation reaches around 550%, it reaches its maximum value, and then sharply decreases, with the increase in stretching elongation.

(2) Testing of Natural 3D Crimp Characteristics

**Figure 19:**
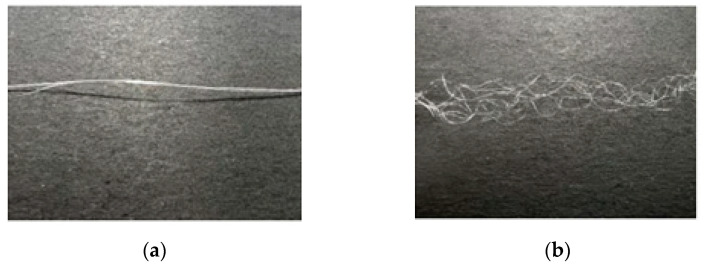
Three-dimensional crimp shape. (**a**) After heat setting; (**b**) After non-heat setting.

The eight-leaf square hollow profiled fibers did not exhibit a three-dimensional crimp shape after the application of heat setting tension, although they exhibited a slightly bent shape. However, after non-heat setting tension application, they exhibited a three-dimensional crimp shape, as shown in Figure 19.

The spinning process parameters are as follows: The single hole volume flow rate is taken as 3.33 × 10^−8^ m^3^/s, the initial blowing height is considered as 80 mm, the spinning temperature is 285 °C, the winding speed is 1200 m/min, and three groups of fibres with cooling speeds of 0.6 m/s, 0.8 m/s, and 1 m/s respectively. By employing a curl flexibility test instrument to test, it is observed that under the same conditions of other spinning process parameters, the average crimp of a single fibre is found to increases with the increase of cooling speed. 

In this section, two complex-shaped units were extruded and bonded together to form a special cross-sectional shape with a hollow structure. Then, eight-leaf square hollow profiled fibers were prepared using the micro-hole design. The internal stresses of every part of the fiber during the spinning process were found to be different; therefore, the fibers showed the characteristics of a natural three-dimensional crimp.

## 5. Conclusion and Discussion

This work focused on the preparation of multi-leaf hollow profiled fibers. The numerical simulation of the extrusion-forming process of multi-leaf fibers was performed. Taking the six-leaf hollow profiled fibers as an example, the characteristics and related preparation methods of the multi-leaf hollow fibers have been discussed. Then, the process of forming the cross-section was studied. The design requirements of the spinning pack and the design method applied to the profiled micro-hole spinnerets were also studied. Further, the finite element method was employed to numerically simulate the melt flow and extrusion swell of the designed spinneret channel. The experiment results reveal that as the volume flow rate of a single hole increased to a certain extent, it promoted the adhesion of the melt. Finally, numerical simulations were performed on the flow of the melt, and the extrusion effect was investigated. Furthermore, we adopt a combination of numerical simulation and experimental verification for the preparation of eight-leaf square hollow profiled fibers, with a 40.99% degree of profile. These fibers not only have complex cross-section shapes but also possess natural three-dimensional crimp characteristics. The mechanisms of formation of these complex multi-leaf hollow profiled fibers require further research. 

With the emerging trend of developing new types of fibers, such as functional fibers, smart fabric, and revolutionary fibers, this paper provides a theoretical reference and experimental foundation for the development of profile fibers. Kapok fiber, ramie fiber, polar bear hair, and other natural fibers with hollow structures have excellent warmth retention and good compression resilience. From the perspective of bionics fiber research and development, these natural characteristics can be reproduced by applying the hollow structure of natural fibers when developing synthetic fibers. Further, hollow fibers are widely used in the textile and clothing industry. However, multi-leaf hollow profiled fibers are still in the experimental exploration stage, and the mechanical properties of the fibers are yet to be sufficiently improved so as to be suitable for industrial production and product applications. Experiments on the preparation of eight-leaf hollow profiled fibers revealed a three-dimensional crimp property after drawing without a heat setting. Further research should be undertaken to enhance the related theoretical research, as well as to ensure improvements in the spinning process and product applications.

## Figures and Tables

**Table 1:** Six-leaf hollow fiber spinning process parameters.

Parameters	Six-Leaf Hollow
Single-hole volume flow rate m^3^/s	4.75 × 10^−8^
Equivalent diameter mm	0.228
Shear rate m·s^−1^	2450.55
Draw-down ratio	265

**Table 2:** Material parameters used in the simulation.

Parameters	Values
Non-Newtonian index *n*	0.69
Relaxation time s	0.012
Zero-shear viscosity Pa·s	20
Limit-shear viscosity Pa·s	120
Density kg·m^−3^	1.268
Thermal conductivity w·m^−1^k^−1^	0.21
Heat capacity J·kg·k^−1^	3.452

**Table 3:** The pressure drop in the spinneret.

Name	Six-Leaf Hollow
Filter screen, MPa	0.691
Distributinon plate, MPa	0.008
Spinneret plate, MPa	6.371
Total pressure drop, MPa	7.07

**Table 4:** Spinning process parameters of six-leaf hollow fiber.

Items		Six-Leaf Hollow
Screw extruder	Temperature of zone 1 (°C)	282
	Temperature of zone 2 (°C)	292
	Temperature of zone 3 (°C)	295
	Temperature of zone 4 (°C)	292
Temperature of gear pump (°C)		285
Temperature of spinning pack (°C)		285
Initial blowing height (mm)		80
Speed of cooling air (m/s)		0, 0.3, 0.6, 0.9
Temperature of cooling air (°C)		24
Single-hole inlet flow (m^3^/s)		4.75 × 10^−8^

**Table 5:** Calculation methods for some parameter of eight-leaf square hollow fiber.

Item	Eight-Leaf Square Hollow
Single-hole volume flow rate, m^3^/s	3.33 × 10^−8^
Equivalent diameter, mm	0.149
Shear rate, m·s^−1^	3468.872
Draw-down ratio	287.04

**Table 6:** Spinning process parameters of eight-leaf square hollow fiber.

Parameters		Values
**Screw extruder**	Temperature of zone 1 (°C)	276
	Temperature of zone 2 (°C)	292
	Temperature of zone 3 (°C)	295
	Temperature of zone 4 (°C)	292
	Temperature of cooling air (°C)	24
Volume flow rate of single hole (m^3^/s)		2.33 × 10^−8^, 2.67 × 10^−8^, 3.00 × 10^−8^ 3.33 × 10^−8^, 3.67 × 10^−8^, 4.00 × 10^−8^ 4.33 × 10^−8^
Speed of cooling air (m/s)		0.8
Initial blowing height (mm)		80
Temperature of spinning pack (°C)		285
Winding speed (m/min)		1200

**Table 7:** Final spinning process parameters of eight-leaf square hollow fiber.

Items	Parameters
Volume flow rate of single hole (m^3^/s)	3.33 × 10^−8^
Speed of cooling air (m/s)	0.8
Initial blowing height (mm)	80
Temperature of spinning pack (°C)	285
Winding speed (m/min)	1200

## Data Availability

The data presented in this study are available upon request from the corresponding author. For data protection reasons, the data are not publicly accessible.

## Supplementary Materials

The following supporting information can be downloaded at: www.mdpi.com/xxx/s1, Figure S1. The cooling speed is 0.6 m/s. (a) Spun fiber cross-section; (b) cross-section of fiber after winding; (c) fiber surface after winding.; Figure S2. The cooling speed is 0.8m/s. (a) Spun fiber cross-section; (b) cross-section of fiber after winding; (c) fiber surface after winding.; Figure S3. The cooling speed is 1.0 m/s. (a) Spun fiber cross-section; (b) cross-section of fiber after winding; (c) fiber surface after winding; Figure S4. The initial blowing height is 80 mm. (a) Spun fiber cross-section; (b) cross-section of fiber after winding; (c) fiber surface after winding.; Figure S5. The initial blowing height is 110 mm. (a) Spun fiber cross-section; (b) cross-section of fiber after winding; (c) fiber surface after winding; Figure S6. The initial blowing height is 140 mm. (a) Spun fiber cross-section; (b) cross-section of fiber after winding; (c) fiber surface after winding.; Figure S7. The spinning temperature is 285 °C. (a) Spun fiber cross-section; (b) cross-section of fiber after winding; (c) fiber surface after winding.; Figure S8. The spinning temperature is 287 °C. (a) Spun fiber cross-section; (b) cross-section of fiber after winding; (c) fiber surface after winding.; Figure S9. The spinning temperature is 290 °C. (a) Spun fiber cross-section; (b) cross-section of fiber after winding; (c) fiber surface after winding.; Figure S10. The winding speed is 1200 m/min. (a) Spun fiber cross-section; (b) cross-section of fiber after winding; (c) fiber surface after winding.; Figure S11. The winding speed is 1500 m/min. (a) Spun fiber cross-section; (b) cross-section of fiber after winding; (c) fiber surface after winding; Figure S12. The winding speed is 1800 m/min. (a) Spun fiber cross-section; (b) cross-section of fiber after winding; (c) fiber surface after winding.; Table S1. Spinning process parameters of eight-leaf square hollow fiber; Table S2. Spinning process parameters of eight-leaf square hollow fiber; TableS3. Spinning process parameters of eight-leaf square hollow fiber; Table S4. Spinning process parameters of eight-leaf square hollow fiber; Table S5. Mechanical properties testing of eight leaf square hollow profiled fibers.
