# Supplementary material for: Numerical Simulation and Experimental Verification of Melt-Spinning Parameters’ Effects on Multi-Leaf Hollow-Profiled Fiber Preparation
Source: Polymers (Basel). 2024 Jan 13;16(2):0. doi: 10.3390/polym16020228 (PMC11154422; doi:10.3390/polym16020228)
Supplement: Supplementary file 1 [file polymers-16-00228-s001.zip › polymers-2698965-supplementary.pdf]

# Supplementary

# Numerical Simulation and Experimental Verification of Melt-Spinning Parameters' Effects on Multi-Leaf Hollow-Profiled Fibre Preparation

**Shiqun He [1], Xinkang Xu [1], Pei Feng [1,2,*], Chongchang Yang [1,2] and Shengze Wang [1]**

**Part 1: This part is the supplementary for section 4.3.**

The experimental results on the effects of cooling speed, initial blowing height, spinning temperature, and winding speed on cross-sectional deformation are as follows:

(1) The influence of cooling speed on the cross-sectional shape

**Table S1.** Spinning process parameters of eight-leaf square hollow fiber.

| Parameters | Values |
|---|---|
| Volume flow rate of single hole($m^3/s$) | $3.33\times10^{-8}$ |
| Speed of cooling air (m/s) | 0.6, 0.8, 1 |
| Initial blowing height (mm) | 80 |
| Temperature of spinning pack (℃) | 285 |
| Winding speed (m/min) | 1200 |

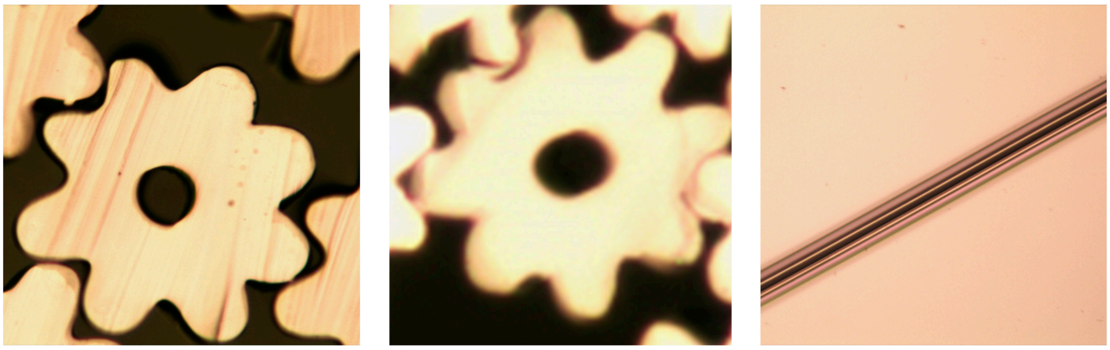

(a) (b) (c)

**Figure S1.** The cooling speed is 0.6m/s. (a) Spun fiber cross-section; (b) cross-section of fiber after winding; (c) fiber surface after winding.

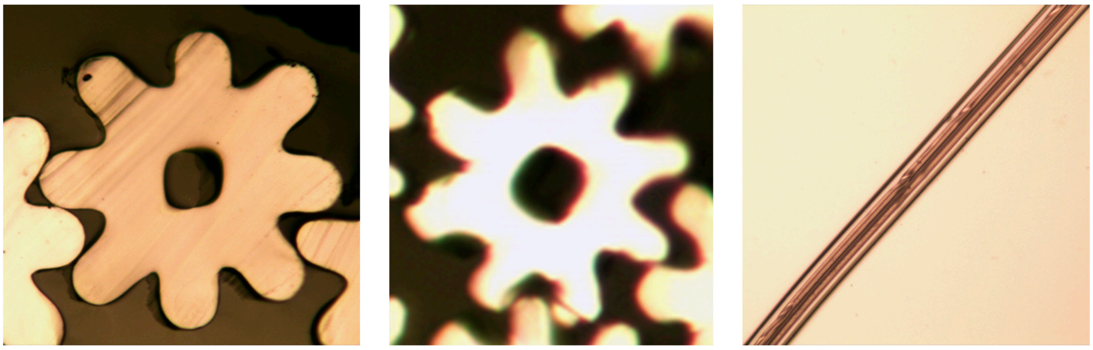

(a) (b) (c)

**Figure S2.** The cooling speed is 0.8m/s. (a) Spun fiber cross-section; (b) cross-section of fiber after winding; (c) fiber surface after winding.

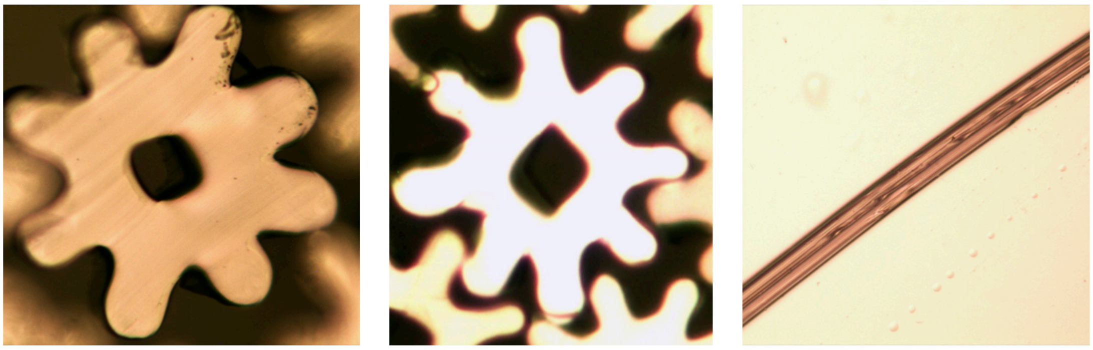

(a) (b) (c)

**Figure S3.** The cooling speed is 1.0 m/s. (a) Spun fiber cross-section; (b) cross-section of fiber after winding; (c) fiber surface after winding.

(2) The influence of initial blowing height on the cross-section shape

**Table S2.** Spinning process parameters of eight-leaf square hollow fiber.

| Parameters | Values |
| --- | --- |
| Volume flow rate of single hole($m^3/s$) | $3.33\times10^{-8}$ |
| Speed of cooling air (m/s) | 0.8 |
| Initial blowing height (mm) | 80,110,140 |
| Temperature of spinning pack (℃) | 285 |
| Winding speed (m/min) | 1200 |

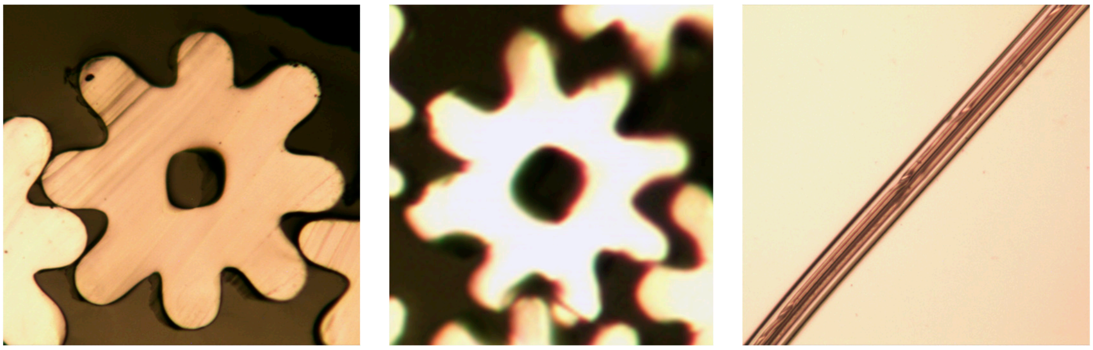

(a) (b) (c)

**Figure S4.** The initial blowing height is 80 mm. (a) Spun fiber cross-section; (b) cross-section of fiber after winding; (c) fiber surface after winding.

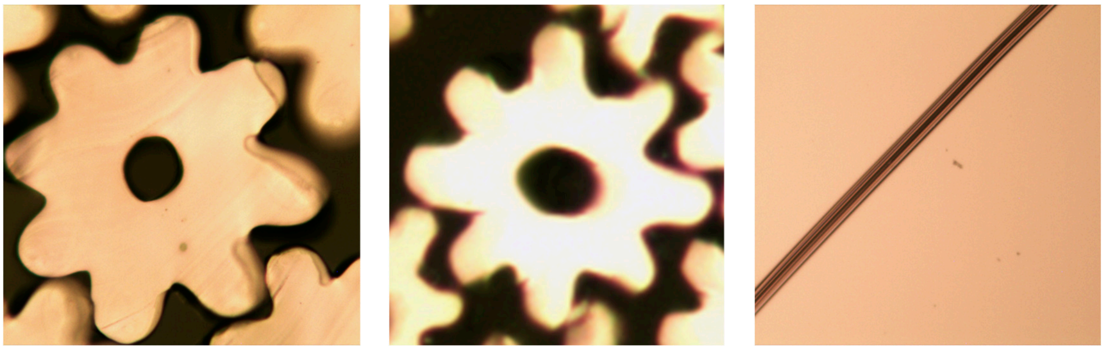

(a) (b) (c)

**Figure S5.** The initial blowing height is 110 mm. (a) Spun fiber cross-section; (b) cross-section of fiber after winding; (c) fiber surface after winding.

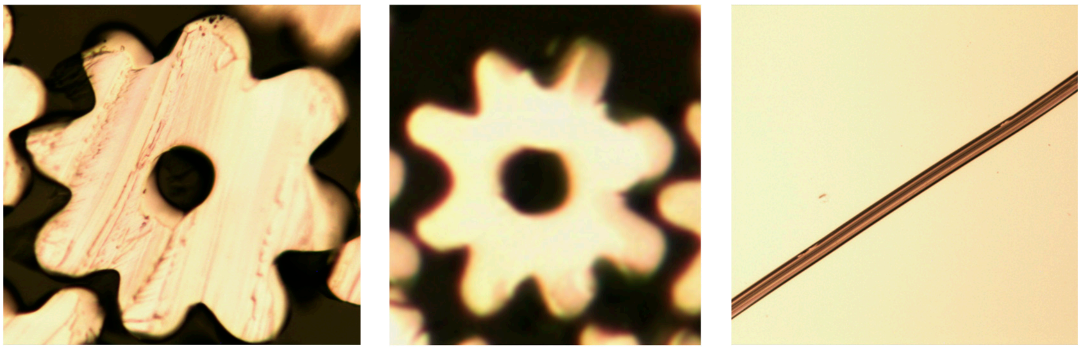

(a) (b) (c)

**Figure S6.** The initial blowing height is 140 mm. (a) Spun fiber cross-section; (b) cross-section of fiber after winding; (c) fiber surface after winding.

(3) The influence of spinning temperatures on the cross-section shape

**Table S3.** Spinning process parameters of eight-leaf square hollow fiber.

| Parameters | Values |
|---|---|
| Volume flow rate of single hole($m^3/s$) | $3.33\times10^{-8}$ |
| Speed of cooling air (m/s) | 0.8 |
| Initial blowing height (mm) | 80 |
| Temperature of spinning pack (°C) | 285,287,290 |
| Winding speed (m/min) | 1200 |

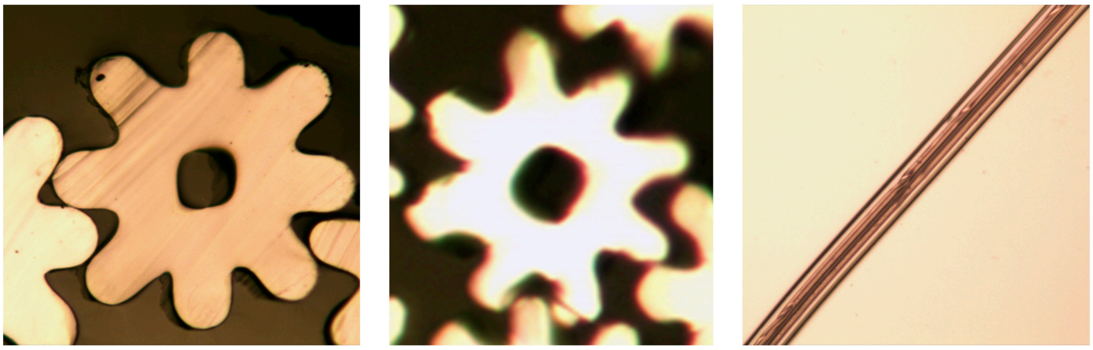

(a) (b) (c)

**Figure S7.** The spinning temperature is 285℃. (a) Spun fiber cross-section; (b) cross-section of fiber after winding; (c) fiber surface after winding.

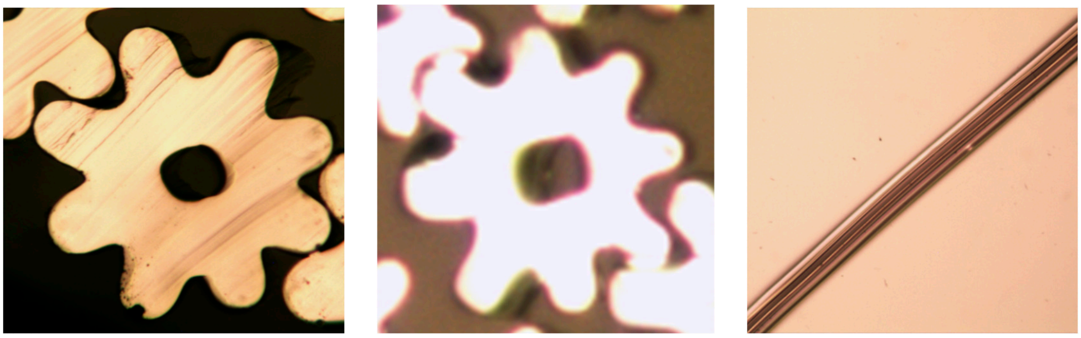

(a) (b) (c)

**Figure S8.** The spinning temperature is 287℃. (a) Spun fiber cross-section; (b) cross-section of fiber after winding; (c) fiber surface after winding.

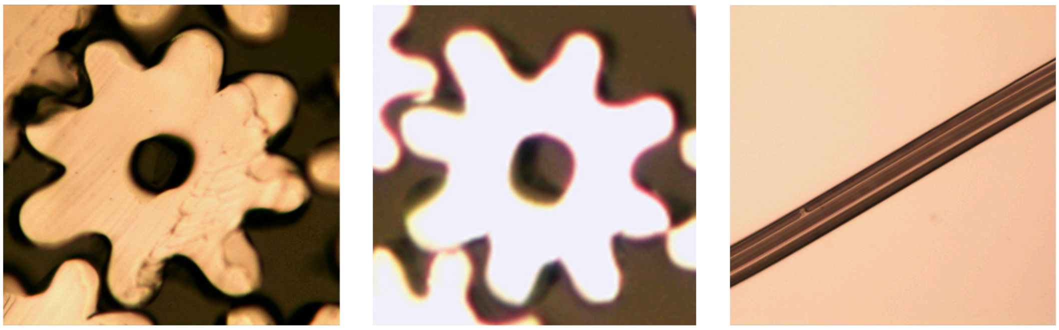

(a) (b) (c)

**Figure S9.** The spinning temperature is 290℃. (a) Spun fiber cross-section; (b) cross-section of fiber after winding; (c) fiber surface after winding.

(4) The influence of winding speed on the cross-section shape

**Table S4.** Spinning process parameters of eight-leaf square hollow fiber.

| Parameters | Values |
|---|---|
| Volume flow rate of single hole($m^3/s$) | $3.33 \times 10^{-8}$ |
| Speed of cooling air (m/s) | 0.8 |
| Initial blowing height (mm) | 80 |
| Temperature of spinning pack (°C) | 285 |
| Winding speed (m/min) | 1200,1500,1800 |

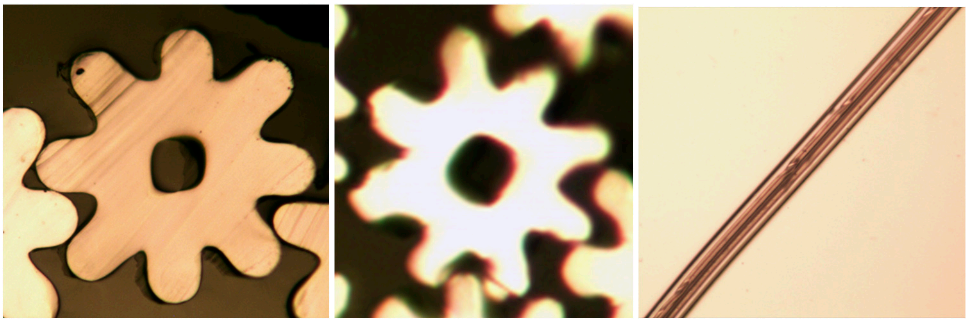

(a) (b) (c)

**Figure S10.** The winding speed is 1200 $m/min$. (a) Spun fiber cross-section; (b) cross-section of fiber after winding; (c) fiber surface after winding.

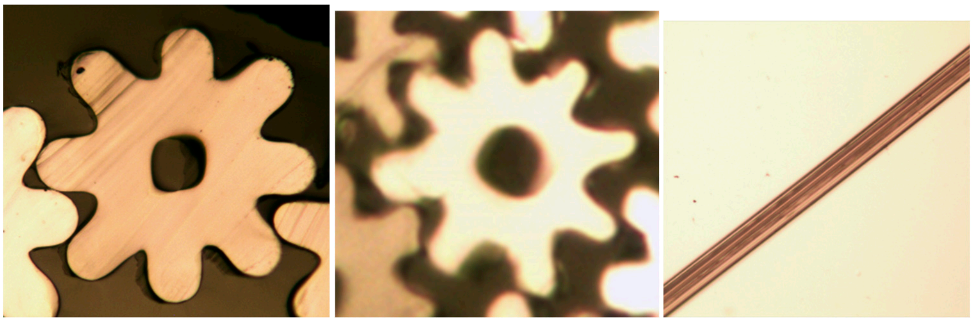

(a) (b) (c)

**Figure S11.** The winding speed is 1500 $m/min$. (a) Spun fiber cross-section; (b) cross-section of fiber after winding; (c) fiber surface after winding.

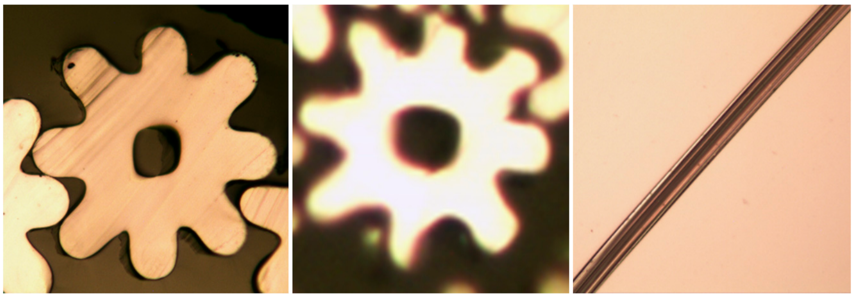

(a) (b) (c)

**Figure S12.** The winding speed is 1800 $m/min$. (a) Spun fiber cross-section; (b) cross-section of fiber after winding; (c) fiber surface after winding.

**Part 2:This part is the supplementary for section 4.4**

Testing of fiber mechanical properties. With linear density is 360dtex, clamping length is 100 mm, drawing speed is 200 $m/min$, ambient temperature is 23℃, ambient humidity is 50%RH, tension coefficient is 0.5. The result of mechanical properties of eight-leaf square hollow profiled fibers is in Table5.

**Table S5.** Mechanical properties testing of eight leaf square hollow profiled fibers.

| Times | Breaking strength cN | Breaking elongation mm | Breaking time s | breaking strength cN/tex |
|---|---|---|---|---|
| 1 | 320 | 549.90 | 165.20 | 8.80 |
| 2 | 291 | 537.30 | 161.30 | 8.00 |
| 3 | 309 | 540.10 | 162.30 | 8.50 |
| 4 | 327 | 560.60 | 168.50 | 9.00 |
| 5 | 309 | 567.90 | 170.50 | 8.50 |
| 6 | 303 | 567.40 | 170.50 | 8.40 |
| 7 | 311 | 543.30 | 163.30 | 8.60 |
| 8 | 282 | 525.20 | 157.80 | 7.80 |
| 9 | 275 | 540.90 | 162.50 | 7.60 |
| 10 | 279 | 581.10 | 174.30 | 7.70 |

| | | | | |
|---|---|---|---|---|
| Maximum | 327 | 581.10 | 174.30 | 9.00 |
| Minimum | 275 | 525.20 | 157.80 | 7.60 |
| Average | 301 | 551.30 | 165.60 | 8.30 |
| CV（%） | 6 | 2.94 | 2.94 | 5.54 |
